# Interpregnancy change in body mass index and infant outcomes in Texas: a population-based study

**DOI:** 10.1186/s12884-019-2265-z

**Published:** 2019-04-05

**Authors:** Renata H. Benjamin, Sarah Littlejohn, Mark A. Canfield, Mary K. Ethen, Fei Hua, Laura E. Mitchell

**Affiliations:** 1 UTHealth School of Public Health, Department of Epidemiology, Human Genetics and Environmental Sciences, 1200 Pressler Street, Houston, TX 77030 USA; 2 Birth Defects Epidemiology and Surveillance Branch, Texas Department of State Health Services, Austin, TX USA; 3 Center for Health Statistics, Texas Department of State Health Services, Austin, TX USA

**Keywords:** Preterm, Small for gestational age, Large for gestational age, Interpregnancy weight change, Body mass index

## Abstract

**Background:**

Maternal prepregnancy body mass index (BMI) is associated with several infant outcomes, but it is unclear whether these associations reflect causal relationships. We conducted a study of interpregnancy change in BMI (IPC-BMI) to improve understanding of the associations between BMI and large for gestational age (LGA), small for gestational age (SGA), and preterm birth (PTB).

**Methods:**

Birth certificate data from 2481 linked sibling pairs (Texas, 2005–2012) were used to estimate IPC-BMI and evaluate its association with LGA, SGA, and PTB in the younger sibling of the pair. Multivariable logistic regression was used to estimate adjusted odds ratios (aOR) and 95% confidence intervals (CI) using data from the full sample and within strata defined by prepregnancy BMI for the older sibling.

**Results:**

On average, women gained 1.1 BMI units between pregnancies. In the full sample, interpregnancy BMI decreases were associated with reduced odds of LGA and increased odds of SGA and PTB (IPC-BMI < -1 versus 0 to < 1: LGA aOR 0.7, 95% CI 0.4, 1.1; SGA aOR 1.6, 95% CI 1.0, 2.7; PTB aOR 1.9, 95% CI 1.3, 2.8). In stratified analyses, similar associations were observed in some, but not all, strata. Findings for interpregnancy BMI increases were less consistent, with little evidence for associations between these outcomes and the most extreme IPC-BMI increases.

**Conclusions:**

There is growing evidence that interpregnancy BMI decreases are associated with LGA, SGA, and PTB. However, taken as a whole, the literature provides insufficient evidence to establish causal links between maternal BMI and these outcomes.

## Background

Maternal prepregnancy weight is associated with a range of infant outcomes. Compared to the offspring of women with normal prepregnancy body mass index (BMI), the offspring of obese women are at higher risk of death, congenital malformation, preterm birth (PTB), and being large for gestational age (LGA) [1]. Whether these associations reflect causal influences or confounding by factors, such as caloric intake and physical activity, has not been established.

Villamor and Cnattingius [2] noted that support for a causal relationship between maternal weight and an infant outcome would be strengthened by evidence that temporal changes in an individual’s weight are also associated with the outcome and suggested change in prepregnancy BMI between successive pregnancies, or interpregnancy change in BMI (IPC-BMI), as a measure of temporal weight change. IPC-BMI has subsequently been associated with LGA, small for gestational age (SGA), and PTB [2–7]. However, the literature on IPC-BMI and these specific outcomes is sparse. Although a recent meta-analysis of IPC-BMI and infant outcomes identified 11 studies of IPC-BMI and pregnancy outcomes, only four studies of LGA and three studies of SGA were included in the meta-analyses [8]. Further, PTB was omitted from the meta-analysis due to a lack of relevant data for calculating a pooled estimate for this outcome [8].

Given the high rates of overweight and obesity in women of reproductive age, research to improve understanding of the association between maternal weight and infant outcomes is essential. Additional studies demonstrating associations between IPC-BMI and infant outcomes would serve to strengthen the evidence for a causal relationship between maternal weight and infant outcome. Hence, we conducted a study of IPC-BMI to improve understanding of the associations between maternal pre-pregnancy BMI and LGA, SGA, and PTB.

## Methods

### Study design and population

We conducted a population-based study of the association between IPC-BMI and three outcomes, LGA, SGA, and PTB, in liveborn, singleton infants. The study was based on a simple random sample of livebirths, without a known chromosomal abnormality or structural birth defect, to Texas residents, 2006–2012. This sample was initially selected as a control population for a study of spina bifida that used a 10:1 ratio of controls to cases. Briefly, infants with a chromosomal abnormality or structural birth defect were identified by linking all birth certificates, 2006–2012, to Texas residents (*N* = 2,759,061) to the Texas Birth Defects Registry. Then, from the 2,623,512 certificates that did not link to the Registry, a simple random sample of 8760 births (10 times the number of spina bifida cases) was selected. Birth certificates for these infants, referred to as index infants, were linked to birth certificates for siblings who were liveborn to Texas residents, 2005–2012. We used data from the birth certificate of the index infant (e.g. maternal first and last name, date of birth, social security number) and probabilistic matching methods to identify birth certificates for the siblings. Matches were generated using Link Plus (Version 3.0 Beta, Atlanta, GA) and manually reviewed for accuracy. The study timeframe was based on data availability: 2005 was the first year that maternal prepregnancy height and weight were recorded on Texas birth certificates and 2012 was the most recent year of completed data at the time of study initiation.

To be eligible for this study, the birth certificate for the index infant had to link to at least one older liveborn sibling. When an index infant was linked to more than one older sibling, only the linked sibling with the date of birth closest to the index infant was retained. Index-sibling pairs were excluded when the index infant was from a multiple birth, information on maternal prepregnancy weight was missing from either birth certificate, or maternal height on the index infant and sibling birth certificates differed by more than 2 inches.

Approval for this study was obtained from the Texas Department of State Health Services Institutional Review Board and the University of Texas Health Science Center at Houston Committee for the Protection of Human Subjects. The requirement to obtain informed consent was waived for this project.

### Study variables

Data for all study variables were obtained from information on the birth certificates. These data come from a variety of sources. For example, data for maternal height and weight are based on maternal self-report, gestational age at birth is based on the obstetrician’s estimate, and data for maternal conditions, such as hypertension, may come from prenatal care or labor and delivery records.

The outcomes considered in this study were LGA, SGA, and PTB in the index infant, and unless noted otherwise, data were obtained from the birth certificate of the index infant. For comparability with prior studies, LGA and SGA were defined as birthweight for gestational age and sex greater than the 90th and less than the 10th percentile, respectively, based on a reference population of livebirths in the United States [9]. PTB was defined as delivery prior to 37 completed weeks of gestation. All outcome definitions were based on the obstetric estimate of gestational age at birth.

Maternal self-reported height and prepregnancy weight from the index infant and sibling birth certificates were used to calculate maternal BMI (kg/m^2^) for the index (BMI-Index) and sibling (BMI-Sib) pregnancies, respectively. When the recorded heights differed, the shorter height was used to calculate both BMI values, since women tend to overestimate their height [10, 11]. When height was missing from one record, height from the other record was used to calculate both BMI values. We calculated IPC-BMI by subtracting BMI-Sib from BMI-Index and expressed the difference as a unit change (e.g. a decrease from 22.0 to 20.5 is a−1.5 unit change).

Gestational weight gain in the index pregnancy was calculated as the difference between maternal weight at delivery and maternal prepregnancy weight and categorized as adequate, inadequate, or excessive based on the Institute of Medicine BMI-specific guidelines for adequate weight gain (in pounds): underweight, 28–40; normal, 25–35; overweight, 15–25; obese, 11–20 [12].

Data from both the index infant and sibling birth certificates were used to classify maternal race/ethnicity. When the recorded race/ethnicity differed on linked birth certificates and one was recorded as non-Hispanic white and the other as a different race/ethnicity, we coded the mother as the non-white category. When two different non-white race/ethnicity categories were recorded, race/ethnicity was categorized as other. The interpregnancy interval was calculated as the difference between the date of birth on the sibling birth certificate and the estimated date of conception (birth date minus the obstetric estimate of gestational age) from the index infant birth certificate.

### Statistical methods

Maternal and infant characteristics were described using counts and frequencies for categorical variables and means and standard deviations for continuous variables. Cells including five or fewer observations were suppressed, along with complementary suppression of another table cell, to protect the identity of study subjects. To assess the association between IPC-BMI and each outcome in the index infants, we estimated adjusted odds ratios (aORs) and 95% confidence intervals (CIs) using multivariable logistic regression.

In the full sample, IPC-BMI was assessed as a six-level variable defined by unit change in BMI: <− 1, − 1 to < 0, 0 to < 1 (reference), 1 to < 2, 2 to < 3, and ≥ 3 units. For consistency with the majority of published studies on IPC-BMI and infant outcomes, we adjusted all estimates for BMI-Sib using standard BMI categories: underweight (< 18.5 kg/m^2^), normal (18.5–24.9 kg/m^2^), overweight (25.0–29.9 kg/m^2^) and obese (≥ 30 kg/m^2^). Estimates of association were also adjusted for the following maternal variables that were selected based on relevant literature: race/ethnicity, gestational weight gain (LGA and SGA only), and smoking. In addition, we evaluated the following possible confounders using a backward elimination strategy: age, education, marital status, payment source, gestational weight gain (PTB analyses only), interpregnancy interval, and maternal height, and retained variables with a likelihood ratio test *P* < 0.05 in the multivariable models.

To assess potential differences in the associations between IPC-BMI and infant outcomes across categories of maternal BMI, we also conducted multivariable logistic regression analyses separately within strata defined by BMI-Sib. For these analyses, IPC-BMI was defined as a three-level, categorical variable: < 0, 0 to < 1 (reference) and ≥ 1. With the exception of BMI-Sib (i.e. the stratification variable), analyses within strata were adjusted for the same covariates used in the analyses of the full sample.

To assess the robustness of our results to study inclusion criteria, we repeated the analyses that were conducted in the full sample in five subsets of index-sibling pairs: (1) pairs without maternal diabetes or hypertensive disorders (pregestational or gestational) recorded on the index birth certificate, (2) pairs with the same father (i.e. the index infant and sibling birth certificates matched on at least two paternal identifiers: date of birth, first, middle or last name), (3) pairs from consecutive livebirths (i.e. with no intervening livebirths), (4) pairs that were the mother’s first and second births, and (5) pairs in which the sibling did not have the outcome of interest. For PTB only, we also repeated our analyses after excluding gestational weight gain as a covariate, since women who deliver preterm may have lower average weight gains due to shorter lengths of gestation than women who deliver at term.

For all statistical tests, *P* < 0.05 was considered statistically significant. Odds ratios with 95% confidence intervals that excluded 1.0 were also considered statistically significant. All analyses were conducted using SAS software version 9.4 (SAS Institute, Cary, NC).

## Results

Out of 8760 randomly selected infants born to Texas residents between 2006 and 2012, the birth certificates for 3349 reported the birth of an older sibling between 2005 and 2012. Of these, 2903 (87%) were linked to a prior live born sibling. Of the linked pairs, we excluded 43 (1.5%) with missing maternal prepregnancy weight on the birth certificate for the index or sibling. An additional 298 (10.3%) linked pairs with a maternal height difference of more than 2 inches were excluded, and 81 (2.8%) linked pairs were excluded because the index infant was from a multiple birth. The final analytic sample included 2481 linked pairs, including 240 (9.7%) LGA, 199 (8.0%) SGA, and 255 (10.3%) preterm index infants.

Characteristics of the index infants and their mothers in the full analytic sample, and in the LGA, SGA, and PTB subsets, are summarized in Table 1. In the full sample, 43% of women were either overweight or obese prior to the sibling pregnancy. This proportion increased to 52% prior to the index pregnancy. Maternal prepregnancy BMI remained relatively stable (i.e. 0 to < 1 unit increase) between the sibling and index pregnancies in 27% of women, increased by one or more units in 43% and decreased in 30%. On average, women gained 1.1 BMI units over an average interpregnancy interval of 21.4 months. Given the relatively small number in the other race/ethnicity category (*N* = 113), we excluded this group from all subsequent analyses.

**Table 1:** Characteristics of index infants and their mothers, Texas 2006–2012

	N^a^ (%) or Mean (SD)			
	All Infants (*N* = 2481)	LGA (*N* = 240)	SGA (*N* = 199)	PTB (*N* = 255)
Mean gestational age (weeks)	38.3 (1.8)	38.0 (2.0)	38.5 (1.6)	34.5 (2.4)
Sex				
Male	1239 (49.9)	129 (53.8)	99 (49.8)	115 (45.1)
Female	1242 (50.1)	111 (46.3)	100 (50.3)	140 (54.9)
Birth order				
2	1176 (48.4)	95 (40.6)	95 (47.7)	102 (41.5)
> 2	1255 (51.6)	139 (59.4)	104 (52.3)	144 (58.5)
Payment method				
Private insurance	852 (34.4)	95 (39.8)	53 (26.6)	72 (28.2)
Medicaid	1229 (49.6)	99 (41.4)	115 (57.8)	143 (56.1)
Self-pay	229 (9.2)	24 (10.0)	17 (8.5)	19 (7.5)
Other	169 (6.8)	21 (8.8)	14 (7.0)	21 (8.2)
Maternal race/ethnicity				
Non-Hispanic White	812 (32.7)	94 (39.2)	49 (24.6)	82 (32.2)
Hispanic	1279 (51.6)	131 (54.6)	98 (49.3)	124 (48.6)
Non-Hispanic Black	277 (11.2)	NR	39 (19.6)	42 (16.5)
Other	113 (4.6)	NR	13 (6.5)	7 (2.8)
Age (years)				
< 20	163 (6.6)	15 (6.3)	14 (7.0)	21 (8.2)
20–24	690 (27.8)	43 (17.9)	63 (31.7)	86 (33.7)
25–29	758 (30.6)	84 (35.0)	72 (36.2)	62 (24.3)
30–34	581 (23.4)	60 (25.0)	34 (17.1)	63 (24.7)
≥ 35	289 (11.7)	38 (15.8)	16 (8.0)	23 (9.0)
Marital status				
Married	1526 (61.5)	165 (68.8)	109 (54.8)	144 (56.5)
Not married	955 (38.5)	75 (31.3)	90 (45.2)	111 (43.5)
Education				
< High school	666 (26.9)	68 (28.3)	64 (32.2)	72 (28.2)
High school	683 (27.5)	68 (28.3)	61 (30.7)	82 (32.2)
> High school	1131 (45.6)	104 (43.3)	74 (37.2)	101 (39.6)
Smoking^b^				
Yes	153 (6.2)	11 (4.6)	17 (8.5)	21 (8.3)
No	2326 (93.8)	229 (95.4)	182 (91.5)	233 (91.7)
Hypertension^c^				
Yes	105 (4.2)	13 (5.4)	10 (5.0)	23 (9.0)
No	2372 (95.8)	227 (94.6)	189 (95.0)	232 (91.0)
Mean interpregnancy interval (months)	21.4 (15.1)	22.1 (15.0)	19.6 (14.4)	19.6 (14.9)
Interpregnancy interval (months)				
< 6	318 (12.8)	25 (10.4)	35 (17.6)	46 (18.0)
6 to < 12	474 (19.1)	40 (16.7)	40 (20.1)	52 (20.4)
12 to < 18	452 (18.2)	50 (20.8)	35 (17.6)	40 (15.7)
≥ 18	1237 (49.9)	125 (52.1)	89 (44.7)	117 (45.9)
Gestational weight gain^d^				
Inadequate	596 (24.1)	32 (13.3)	69 (34.7)	88 (34.5)
Adequate	841 (34.0)	78 (32.5)	73 (36.7)	96 (37.7)
Excessive	1040 (42.0)	130 (54.2)	57 (28.6)	71 (27.8)
Height (m)				
< 1.60	1038 (41.8)	80 (33.3)	88 (44.2)	117 (45.9)
1.60 to < 1.65	699 (28.2)	58 (24.2)	66 (33.2)	68 (26.7)
1.65 to < 1.70	434 (17.5)	54 (22.5)	32 (16.1)	42 (16.5)
≥ 1.70	310 (12.5)	48 (20.0)	13 (6.5)	28 (11.0)
Mean prepregnancy BMI - sibling pregnancy (kg/m^2^)	25.6 (5.8)	27.7 (6.2)	25.4 (5.7)	25.2 (5.8)
Prepregnancy BMI - sibling pregnancy (kg/m^2^)				
Underweight (< 18.5)	99 (4.0)	NR	10 (5.0)	16 (6.3)
Normal (18.5–24.9)	1304 (52.6)	97 (40.4)	101 (50.8)	135 (52.9)
Overweight (25.0–29.9)	618 (24.9)	NR	50 (25.1)	54 (21.2)
Obese (≥30.0)	460 (18.5)	72 (30.0)	38 (19.1)	50 (19.6)
Mean prepregnancy BMI - index pregnancy (kg/m^2^)	26.7 (6.3)	28.9 (6.7)	26.3 (6.1)	26.2 (6.6)
Prepregnancy BMI - index pregnancy (kg/m^2^)				
Underweight (< 18.5)	64 (2.6)	NR	NR	9 (3.5)
Normal (18.5–24.9)	1135 (45.8)	80 (33.3)	99 (49.8)	131 (51.4)
Overweight (25.0–29.9)	676 (27.3)	NR	NR	56 (22.0)
Obese (≥30.0)	606 (24.4)	85 (35.4)	47 (23.6)	59 (23.1)
Mean interpregnancy change in BMI (kg/m^2^)	1.1 (3.3)	1.2 (3.7)	0.8 (3.6)	1.0 (3.7)
Interpregnancy change in BMI (kg/m^2^)				
< −1	430 (17.3)	38 (15.8)	45 (22.6)	64 (25.1)
< −1 to < 0	305 (12.3)	24 (10.0)	26 (13.1)	33 (12.9)
0 to < 1	677 (27.3)	64 (26.7)	41 (20.6)	60 (23.5)
1 to < 2	319 (12.9)	37 (15.4)	27 (13.6)	35 (13.7)
2 to < 3	224 (9.0)	31 (12.9)	24 (12.1)	16 (6.3)
≥ 3	526 (21.2)	46 (19.2)	36 (18.1)	47 (18.4)

*Abbreviations*: *BMI* body mass index (kg/m^2^), *LGA* large for gestational age, *NR* not reported due to small cell size and complementary suppression, *PTB* preterm birth, *SD* standard deviation, *SGA* small for gestational age

^a^Numbers may not sum to the total due to missing values

^b^Any smoking during the three months before or during pregnancy

^c^Pregestational or gestational

^d^Per the Institute of Medicine BMI-specific guidelines for adequate weight gain (in pounds): underweight, 28–40; normal, 25–35; overweight, 15–25; obese, 11–20 [12]

Mean IPC-BMI values, within strata defined by maternal prepregnancy BMI at the sibling pregnancy, are summarized in Table 2. In the full sample, women who were underweight at the sibling pregnancy had the highest average IPC-BMI (+ 1.68 units), while women who were obese had the lowest (+ 0.27 units). Similar patterns were observed within the LGA, SGA, and PTB subsets.

**Table 2:** Mean IPC-BMI at sibling pregnancy, Texas 2006–2012

	Mean IPC-BMI (SD), N			
Prepregnancy BMI at Sibling Pregnancy (kg/m^2^)	All Infants	LGA	SGA	PTB
Underweight (< 18.5)	+ 1.68 (2.30), 95	+ 3.02 (1.61), NR	+ 1.95 (2.74), 10	+ 1.77 (1.66), 16
Normal (18.5–24.9)	+ 1.42 (2.83), 1223	+ 1.62 (3.03), 96	+ 1.09 (2.82), 92	+ 1.45 (3.48), 130
Overweight (25.0–29.9)	+ 1.07 (3.63), 599	+ 1.18 (2.93), NR	+ 1.09 (3.71), 47	+ 0.49 (4.06), 52
Obese (≥30.0)	+ 0.27 (4.27), 451	+ 0.50 (4.98), 71	−0.47 (4.56), 37	+ 0.32 (4.51), 50
All	+ 1.12 (3.37), 2368	+ 1.17 (3.71), 237	+ 0.83 (3.49), 186	+ 1.04 (3.77), 248

*Abbreviations*: *BMI* body mass index (kg/m^2^), *IPC-BMI* interpregnancy change in body mass index (kg/m^2^), *LGA* large for gestational age, *NR* not reported due to small cell size and complementary suppression, *PTB* preterm birth, *SD* standard deviation, *SGA* small for gestational age

Results from our analyses of the full sample and within strata defined by maternal prepregnancy BMI at the sibling pregnancy are summarized in Tables 3 and 4, and are described below. Given the small number of infants with LGA, SGA, and PTB in the underweight stratum (*N* ≤ 9, Table 1), this group was omitted from the stratified analyses. Estimates of association were adjusted for BMI-Sib (full sample), maternal race/ethnicity, gestational weight gain, maternal smoking, and variables that were significant in backwards selection: maternal height (LGA and SGA), maternal age (LGA), and maternal education (LGA). The IPC-BMI category of 0 to < 1 served as the reference for all comparisons.

**Table 3:** Associations between IPC-BMI and index infant outcomes, Texas 2006–2012

	LGA			SGA			PTB		
	N^a^	aOR^b^	95% CI	N^a^	aOR^c^	95% CI	N^a^	aOR^d^	95% CI
IPC-BMI	237/2121			186/2173			247/2116		
<−1	38/368	0.70	(0.45, 1.09)	39/367	1.65	(1.01, 2.68)	61/345	1.88	(1.26, 2.81)
−1 to < 0	24/267	0.68	(0.41, 1.14)	25/266	1.52	(0.89, 2.59)	32/259	1.20	(0.76, 1.90)
0 to < 1	63/574	1.00	(Reference)	38/600	1.00	(Reference)	60/579	1.00	(Reference)
1 to < 2	36/270	1.25	(0.80, 1.96)	27/279	1.47	(0.88, 2.48)	34/272	1.20	(0.76, 1.88)
2 to < 3	30/180	1.47	(0.91, 2.39)	23/187	1.91	(1.10, 3.31)	13/198	0.62	(0.33, 1.16)
≥3	46/462	0.92	(0.61, 1.40)	34/474	1.04	(0.64, 1.70)	47/463	0.97	(0.64, 1.46)

*Abbreviations*: *aOR* adjusted odds ratio, *CI* confidence interval, *IPC-BMI* interpregnancy change in body mass index (kg/m^2^), *LGA* large for gestational age, *PTB* preterm birth, *SGA* small for gestational age

^a^N, # affected / # unaffected

^b^Adjusted for the following maternal variables: prepregnancy BMI at sibling pregnancy, race/ethnicity, smoking, gestational weight gain, height, age, and education

^c^Adjusted for the following maternal variables: prepregnancy BMI at sibling pregnancy, race/ethnicity, smoking, gestational weight gain, and height

^d^Adjusted for the following maternal variables: prepregnancy BMI at sibling pregnancy, race/ethnicity, smoking, and gestational weight gain

**Table 4:** IPC-BMI and index infant outcomes within maternal prepregnancy BMI strata at sibling pregnancy, Texas 2006–2012

Outcome	IPC-BMI	Prepregnancy BMI at Sibling Pregnancy								
		Normal (18.5–24.9 kg/m^2^)			Overweight (25.0–29.9 kg/m^2^)			Obese (≥30.0 kg/m^2^)		
		N^a^	aOR	95% CI	N^a^	aOR	95% CI	N^a^	aOR	95% CI
LGA^b^		96/1120			68/529			71/379		
	< 0	18/273	0.64	(0.35, 1.16)	16/189	0.42	(0.19, 0.92)	28/161	1.27	(0.59, 2.76)
	0 to < 1	36/354	1.00	(Reference)	16/105	1.00	(Reference)	11/77	1.00	(Reference)
	≥1	42/493	0.86	(0.53, 1.39)	36/235	1.01	(0.52, 1.97)	32/141	2.13	(0.97, 4.68)
SGA^c^		92/1125			47/550			NR/413		
	< 0	31/260	2.04	(1.14, 3.64)	15/190	0.86	(0.36, 2.09)	17/172	2.20	(0.69, 6.97)
	0 to < 1	22/369	1.00	(Reference)	9/112	1.00	(Reference)	NR/84	1.00	(Reference)
	≥1	39/496	1.31	(0.76, 2.27)	23/248	0.99	(0.44, 2.27)	16/157	1.96	(0.61, 6.29)
PTB^d^		129/1091			52/545			50/401		
	< 0	41/250	1.87	(1.13, 3.07)	25/180	1.51	(0.69, 3.28)	26/163	0.96	(0.45, 2.06)
	0 to < 1	32/359	1.00	(Reference)	10/111	1.00	(Reference)	12/77	1.00	(Reference)
	≥1	56/482	1.35	(0.85, 2.15)	17/254	0.71	(0.31, 1.61)	12/161	0.40	(0.16, 0.95)

*Abbreviations*: *aOR* adjusted odds ratio, *BMI* body mass index (kg/m^2^), *CI* confidence interval, *IPC-BMI* interpregnancy change in body mass index (kg/m^2^), *LGA* large for gestational age, *NR* not reported due to small cell size and complementary suppression, *PTB* preterm birth, *SGA* small for gestational age

^a^N, # affected / # unaffected

^b^Adjusted for the following maternal variables: race/ethnicity, smoking, gestational weight gain, height, age, and education

^c^Adjusted for the following maternal variables: race/ethnicity, smoking, gestational weight gain, and height

^d^Adjusted for the following maternal variables: race/ethnicity, smoking, and gestational weight gain

### LGA

In the full sample, the odds of LGA were lower in the index infants of women with interpregnancy BMI decreases and higher in those with small (IPC-BMI: 1 to < 2) to moderate (IPC-BMI: 2 to < 3) increases. However, the odds of LGA were not increased in the highest IPC-BMI category (IPC-BMI ≥ 3: aOR 0.92, 95% CI 0.61, 1.40) and none of the associations were statistically significant. In the stratified analyses, similar patterns of associations were observed in some, but not all, stratum. Specifically, the odds of LGA with an interpregnancy BMI decease were lower in the normal (aOR 0.64, 95% CI 0.35, 1.16) and overweight (aOR 0.42, 95% CI 0.19, 0.92), but not the obese (aOR 1.27, 95% CI 0.59, 2.76) strata. In contrast, the odds of LGA with an interpregnancy BMI increase were higher only in the obese stratum (aOR 2.13, 95% CI 0.97, 4.68).

### SGA

In the full sample, the odds of SGA were significantly increased in the infants of women with interpregnancy BMI decreases of more than one BMI unit and in the infants of women with moderate BMI increases. Odds were also increased, although not significantly so, in the offspring of women with more moderate losses (IPC-BMI: -1 to < 0) and more moderate gains (IPC-BMI: 1 to < 2). However, SGA was not associated with more extreme increases in BMI (IPC-BMI ≥ 3: aOR 1.04, 95% CI 0.64, 1.70). In stratified analyses, the patterns of associations in the normal and obese strata were similar to those observed in the full sample: increased odds for SGA were observed for the infants of women with either a BMI increase or decrease. However, there was little to no evidence of associations between SGA and IPC-BMI in the overweight stratum.

### PTB

The odds of PTB were highest in the infants of women with an interpregnancy BMI decrease of more than one unit (aOR 1.88, 95% CI 1.26, 2.81) and lowest in those with an increase of 2 to less than 3 units (aOR 0.62, CI 0.33, 1.16). However, the association between PTB and more extreme increases in BMI was close to the null. In stratified analyses, the pattern of association of PTB and IPC-BMI differed across strata. An interpregnancy BMI decrease was associated with increased odds of PTB in the normal (aOR 1.87, 95% CI 1.13, 3.07) and overweight (aOR 1.51, 95% CI 0.69, 3.28), but not the obese (aOR 0.96, 95% CI 0.45, 2.06) strata. In addition, an interpregnancy BMI increase was associated with increased odds of PTB in the normal category (aOR 1.35, 95% CI 0.85, 2.15), and decreased odds in the overweight (aOR 0.71, 95% CI 0.31, 1.61) and obese (aOR 0.40, 95% CI 0.16, 0.95) categories.

### Sensitivity analyses

In general, the direction and magnitudes of the associations observed in the sensitivity analyses were similar to those observed in the analysis of the full sample (Table 5). As in the analysis of the full sample, no significant associations were detected for LGA in any of the sensitivity analyses. For SGA, the positive association with moderate increases in BMI (IPC-BMI 2 to < 3, aOR 1.91, 95% CI 1.10, 3.31) observed in the full sample remained significant in all sensitivity analyses (aOR range: 1.85–2.55). The positive association with loss of more than one BMI unit (aOR 1.65, 95% CI 1.01, 2.68) observed in the full sample, was also observed in all sensitivity analyses (aOR range: 1.28–1.98), although it was statistically significant in only two of these analyses (i.e. consecutive births and outcome not present in the sibling). The positive association between PTB and loss of more than one BMI unit (aOR 1.88, 95% CI 1.26, 2.81) observed in the full sample, was also observed in all sensitivity analyses (aOR range: 1.46–4.05) and remained statistically significant in all but one (outcome not present in sibling) of these analyses. This association was strongest in the sensitivity analysis restricted to include only sibling pairs that were the first and second born (aOR 4.05, 95% CI 1.86, 8.80), suggesting that weight loss may be a stronger risk factor for PTB in earlier, as compared to later, births.

**Table 5:** Sensitivity analyses for the associations between IPC-BMI and infant outcomes, Texas 2006–2012

Outcome	IPC-BMI	No Diabetes or HTN^a^ N^d^		Same Father N^d^		Consecutive Births^b^ N^d^		1st and 2nd Births N^d^		Outcome Not Present in Sibling N^d^		Excluding GWG^c^ N^d^	
		aOR	95% CI	aOR	95% CI	aOR	95% CI	aOR	95% CI	aOR	95% CI	aOR	95% CI
LGA^e^		205/1980		185/1462		206/1797		86/893		167/1983			
	<−1	0.80	(0.50, 1.28)	0.69	(0.41, 1.14)	0.84	(0.52, 1.36)	0.82	(0.37, 1.79)	0.78	(0.47, 1.30)		
	−1 to < 0	0.56	(0.32, 1.01)	0.67	(0.38, 1.18)	0.84	(0.49, 1.43)	0.97	(0.43, 2.21)	0.63	(0.34, 1.17)		
	0 to < 1	1.00	(Reference)	1.00	(Reference)	1.00	(Reference)	1.00	(Reference)	1.00	(Reference)		
	1 to < 2	1.19	(0.74, 1.92)	1.26	(0.76, 2.08)	1.33	(0.81, 2.19)	1.30	(0.58, 2.89)	1.14	(0.66, 1.97)		
	2 to < 3	1.49	(0.90, 2.48)	1.28	(0.72, 2.25)	1.81	(1.07, 3.07)	1.56	(0.69, 3.53)	1.49	(0.85, 2.61)		
	≥3	0.96	(0.61, 1.49)	0.79	(0.49, 1.27)	1.07	(0.69, 1.68)	1.16	(0.58, 2.34)	1.07	(0.67, 1.70)		
SGA^f^		176/2010		109/1539		158/1846		82/897		140/1987			
	<−1	1.51	(0.91, 2.49)	1.50	(0.77, 2.92)	1.92	(1.14, 3.22)	1.28	(0.61, 2.69)	1.98	(1.11, 3.52)		
	−1 to < 0	1.56	(0.92, 2.67)	1.63	(0.81, 3.27)	1.54	(0.87, 2.72)	1.05	(0.47, 2.31)	1.60	(0.84, 3.06)		
	0 to < 1	1.00	(Reference)	1.00	(Reference)	1.00	(Reference)	1.00	(Reference)	1.00	(Reference)		
	1 to < 2	1.49	(0.88, 2.50)	1.90	(0.98, 3.69)	1.34	(0.75, 2.38)	0.72	(0.31, 1.72)	1.87	(1.02, 3.44)		
	2 to < 3	1.85	(1.05, 3.24)	2.11	(1.03, 4.33)	2.10	(1.16, 3.82)	2.31	(1.11, 4.80)	2.55	(1.36, 4.81)		
	≥3	0.92	(0.55, 1.53)	1.33	(0.71, 2.50)	0.91	(0.53, 1.57)	0.65	(0.31, 1.36)	1.52	(0.87, 2.66)		
PTB^g^		220/1970		157/1494		202/1805		86/896		181/1957		247/2116	
	<−1	1.82	(1.20, 2.78)	1.84	(1.12, 3.02)	1.94	(1.25, 3.03)	4.05	(1.86, 8.80)	1.46	(0.92, 2.33)	1.83	(1.23, 2.72)
	−1 to < 0	1.16	(0.72, 1.87)	0.95	(0.53, 1.70)	1.34	(0.82, 2.19)	2.56	(1.12, 5.83)	1.12	(0.67, 1.88)	1.25	(0.79, 1.98)
	0 to < 1	1.00	(Reference)	1.00	(Reference)	1.00	(Reference)	1.00	(Reference)	1.00	(Reference)	1.00	(Reference)
	1 to < 2	1.11	(0.69, 1.78)	1.08	(0.61, 1.91)	1.07	(0.64, 1.78)	2.45	(1.07, 5.60)	1.19	(0.72, 1.97)	1.24	(0.79, 1.94)
	2 to < 3	0.63	(0.33, 1.20)	0.66	(0.31, 1.41)	0.58	(0.28, 1.22)	0.92	(0.29, 3.00)	0.60	(0.29, 1.21)	0.64	(0.34, 1.19)
	≥3	0.85	(0.55, 1.31)	1.12	(0.68, 1.84)	0.97	(0.62, 1.53)	1.95	(0.90, 4.24)	0.82	(0.51, 1.31)	1.00	(0.67, 1.49)

*Abbreviations*: *aOR* adjusted odds ratio, *CI* confidence interval, *GWG* gestational weight gain, *HTN* hypertension, *IPC-BMI* interpregnancy change in body mass index (kg/m^2^), *LGA* large for gestational age, *PTB* preterm birth, *SGA* small for gestational age

^a^Pregestational or gestational diagnosis

^b^Analyses adjusted for birth order in addition to the covariates noted for each outcome

^c^PTB analysis only

^d^N, # affected / # unaffected

^e^Adjusted for the following maternal variables: prepregnancy BMI at sibling pregnancy, race/ethnicity, smoking, gestational weight gain, height, age, and education

^f^Adjusted for the following maternal variables: prepregnancy BMI at sibling pregnancy, race/ethnicity, smoking, gestational weight gain, and height

^g^Adjusted for the following maternal variables: prepregnancy BMI at sibling pregnancy, race/ethnicity, smoking, and gestational weight gain

## Discussion

We conducted a study of IPC-BMI and infant outcomes because studies of BMI change will help to establish whether the observed associations between maternal prepregancy BMI and infant outcomes reflect underlying causal relationships.

Consistent with several prior studies [2, 5, 13], our results indicate that the interpregnancy interval is characterized by weight gain. On average, in our sample, women gained 1.1 BMI units or approximately six pounds (2.7 kg) between the sibling and index pregnancies. Nonetheless, approximately 30% of women lost weight during the interpregnancy interval, and our analyses suggest that such decreases are associated with reduced odds of LGA and increased odds of SGA and PTB. In the full sample, the directions of these associations were consistent across the two BMI loss categories (<− 1 and − 1 to 0), and the magnitudes of the associations were either similar across categories or stronger in the highest BMI loss category. However, our stratified analyses provide some evidence that these associations differ across groups defined by maternal prepregnancy BMI at the initial (sibling) pregnancy. In contrast with our findings for interpregnancy BMI decreases, our analyses provided less support for associations between these infant outcomes and interpregnancy BMI increases.

The literature on IPC-BMI and infant outcomes is sparse and comparisons across studies are hampered by differences in both study populations and study methods. For example, there are differences in the distribution of maternal pre-pregnancy BMI across populations: in our sample, the proportion of women in the overweight and obese categories was ~ 50%, whereas in a Swedish study of IPC-BMI, this proportion was less than 30% [7]. In addition, studies of IPC-BMI have used different measures of BMI change and different subgroup definitions [8]. In some studies, change has been defined with respect to standard BMI categories [14–16], whereas in others it has been defined by categories of unit change in BMI [2–5]. Further, studies that have assessed unit changes have differed in their categorization of this measure, used different reference categories (e.g. <− 1 to 1 BMI units [2, 4]; − 2 to < 2 units [3, 5]) and have not provided the rationale underlying the categorization schemes. In the absence of an established standard, we used the IPC-BMI category of 0 to < 1 units as our reference because it is consistent with the expected BMI trajectory (i.e. increasing BMI with age) [17–19].

Despite differences in the reference groups employed, our results for interpregnancy BMI decreases are generally consistent with prior studies. In particular, our findings of decreased odds of LGA and increased odds of SGA are consistent with meta-analyses of prior studies (LGA summary OR 0.70, 95% CI 0.55, 0.90; SGA summary OR 1.31, 95% CI 1.06, 1.63) [8]. In addition, our finding of increased odds of PTB with interpregnancy BMI decreases are consistent with findings from several [20–22], although not all [5, 15], studies of this association. In stratified analyses, we observed differences in the direction of associations with BMI decreases across the overweight and obese categories (e.g. LGA: overweight, aOR 0.42, 95% CI 0.19, 0.92; obese, aOR 1.27, 95% CI 0.59, 2.76), suggesting that these categories should be considered separately in future studies. Prior studies that conducted stratified analyses combined overweight and obese women [2, 4, 5, 7, 21, 23].

The results of our analyses of interpregnancy BMI increase and both LGA and SGA are difficult to interpret because small to moderate BMI increases were associated with increased odds of both outcomes, whereas more extreme increases were not. In general, prior studies, as well as a meta-analysis, indicate that the odds of LGA increase with interpregnancy BMI increases (meta-analysis summary ORs: IPC-BMI 1–3 units, OR 1.43, 95% CI 1.29, 1.59; IPC-BMI > 3 units, OR 1.85, 95% CI 1.71, 2.00) and that the odds of SGA decrease with BMI increases (meta-analysis summary OR 0.83, 95% CI 0.70, 0.99) [8]. We found little evidence of an association between PTB and interpregnancy BMI increase, which is consistent with the majority of studies that have combined data from both medically-indicated and spontaneous PTB [5, 15, 20, 21].

### Study limitations

Our study required the linkage of sibling birth certificates and is thus limited by the incompleteness of those linkages. Of the potential index infants with at least one prior born sibling (as reported on the birth certificate), 13% were not linked to a prior born sibling. Failure to link may be due to inaccurate or incomplete information on the index or sibling birth certificate, or change in maternal state of residence between deliveries (i.e. a sibling born to a woman who resided outside of Texas at the time of the sibling delivery would not be identified by our linkage strategy). Our study is also limited by the variables included on the certificates and the accuracy of the collected data [24, 25]. Since the Texas birth certificate does not differentiate between spontaneous and medically-indicated PTB, our analyses used data from both categories, which may have obscured associations. This possibility seems likely given that interpregnancy BMI increases have been associated with increased odds of medically-indicated [7, 22], and decreased odds of spontaneous PTB [4, 16, 22]. In addition, birth certificate data for maternal height and weight are based on maternal self-report. Although such data have been shown to be generally valid [10], they are subject to reporting errors that could differ by infant outcome and maternal BMI categories [26].

An additional limitation of this study is the relatively small numbers within strata defined by maternal prepregnancy BMI. Specifically, we were not able to include the underweight group in our stratified analyses, and we were limited to the evaluation of broad exposure categories (e.g. IPC-BMI ≥ 1) in the remaining strata. Further, even with these restrictions, some categories were small and the estimated measures of association were imprecise.

### Study strengths

This study also had several strengths. The study was population-based and included data from relatively recent birth years. In many respects, our study sample appears to be representative of Texas and the United States as a whole. Specifically, the distribution of maternal pre pregnancy BMI in our sample is similar to that reported for all births in Texas and for births across the United States (2014 birth certificate data from 47 states and the District of Columbia) [27], with less than 5% of women in the underweight category and approximately 50% in the overweight and obese categories. In addition, among the index infants, the proportion born premature (10.3%) is consistent with the PTB rate in Texas, which has historically been higher than the rate for the United States as a whole. For example, in 2012 the percent of births that were premature in Texas and in the United States were 10.5 and 9.8%, respectively [28]. The proportion of LGA infants (9.7%) in our sample is also consistent with expectations (10%) based on national standards. Finally, although the proportion of SGA infants (8.0%) is somewhat lower than expected (10%), this is likely to reflect the exclusion of first births, which are at higher risk for SGA than are infants with higher birth orders [29], from the sample (i.e. by definition, index infants had a birth order of at least 2).

We also conducted several sensitivity analyses, which indicated that our findings were unlikely to be significantly affected by our study inclusion criteria. In addition, we addressed differences in maternal height on the sibling and index birth certificates by excluding pairs when this difference was more than 2 inches, and otherwise using the lower of the two reported heights. Most prior studies of IPC-BMI have not described how discrepant information for maternal height was handled. Finally, in stratified analyses, we assessed the overweight and obese categories separately, and found some potentially important differences across these categories.

## Conclusions

Our analyses indicate that interpregnancy decreases in BMI are associated with LGA, SGA, and PTB and, thus, provide some additional evidence for causal relationships between these outcomes and maternal BMI. However, these analyses provide less consistent evidence that BMI increases are associated with these outcomes and, further, suggest that any impact of IPC-BMI may vary across BMI categories. Taken together with the findings from other studies, we conclude that the evidence regarding interpregnancy BMI change is currently insufficient to establish a causal link between BMI and LGA, SGA, or PTB. Consequently, the available data are insufficient for the purposes of making weight-change recommendations specifically for the purpose of reducing the risk of these outcomes. However, given the known health risks of overweight and obesity, general recommendations to achieve and maintain a healthy weight prior to pregnancy (e.g. [30]) remain appropriate.

To help clarify the associations between IPC-BMI and infant outcomes, we support recommendations regarding the need to establish standard measures of BMI change and sub-group definitions [8]. We recommend the use of BMI units as the measure of change, as it is likely to be a more sensitive measure than changes across BMI categories. In addition, we suggest the use of 0 to < 1 units as a reference, because the typical BMI trajectory is characterized by increases over time. Further, our analyses indicate that associations with small unit decreases (− 1 to < 0) are generally similar to those for more extreme decreases (<− 1), such that inclusion of the former in the reference category could obscure associations with weight loss. We also recommend that overweight and obese categories should be differentiated in stratified analyses, since our analyses suggest that there may be differences between these groups. Finally, given potential differences across BMI categories, we advise caution in the interpretation of analyses that are based on combined data as well as consideration of population differences in BMI distributions when comparing results across studies.

## Abbreviations

aOR: Adjusted odds ratio
BMI: Body mass index
CI: Confidence interval
IPC-BMI: Interpregnancy change in body mass index
LGA: Large for gestational age
NR: Not reported
PTB: Preterm birth
SD: Standard deviation
SGA: Small for gestational age
